# Impact of open-air dumping of urban solid waste on soil toxicity and properties in a tropical environment

**DOI:** 10.1007/s11356-026-37862-1

**Published:** 2026-07-03

**Authors:** Ana C. De la Parra-Guerra, Kelly J. Rodelo-Soto, Shania Polo-Camargo, Mildreth Pallares-Arévalo, Erico Marlon Moraes Flores, Rochele Picoloto, Cristian Rafael Andriolli, Jorge Osorio-Martínez, Katy Rematoza-Chamorro, Fabio Fuentes-Gandara

**Affiliations:** 1 Department of Natural and Exact Sciences, Universidad de la Costa, Barranquilla, Colombia; 2 Department of Biology, Universidad del Atlántico, Barranquilla, Colombia; 3 Department of Environmental and Sanitary Engineering, Universidad Popular del Cesar, Aguachica, Colombia; 4 Department of Chemistry, Universidade Federal de Santa Maria, Santa Maria, Brazil; 5 Department of Food Engineering, Universidad de Córdoba, Monteria, Colombia; 6 AMDAC Research Group, José María Córdoba Educational Institution, Monteria Secretary of Education, Monteria, 230017, Colombia

**Keywords:** Open dumping, Toxicity, Deterioration, Leachates, Tropical dry forest, Sustainable

## Abstract

Open dumping (OD) is uncontrolled dumping of solid waste (SW) in an environment. It involves the illegal practice of dumping waste in areas not designated for waste management, such as open lands, flowing water bodies, or exposed waste piles. OD can contain a complex mixture of organic fractions, and various contaminants that, when infiltrated into the soil through leachate, compromise its stability and resistance. These contaminants can reach aquatic ecosystems and natural resources through runoff. In this study, the impact of OD on soil and sediments toxicity and properties in the Caribbean region of Colombia, specifically in a tropical dry forest (Td-F) environment, was evaluated. A characterization of samples was performed, assessing their chemical composition, concentration of trace elements, physical properties, toxicity, and associated risks. The physicochemical conditions were determined using standardized methodologies, while trace elements were determined by inductively coupled plasma mass spectrometry (ICP-MS) after a microwave-assisted digestion method. Additionally, toxicological bioassays were carried out with the *Caenorhabditis elegans* biological model. Soil and sediments samples were collected at nine sampling points distributed throughout the area, at a depth of 20 cm, and weighing approximately 1 kg. The results suggest that the soil in the OD exhibits slight to moderate deterioration associated with the continuous accumulation of SW. The results reveal soil with organic matter (OM) (3 to 8%), density of 2.0 to 2.44 g cm⁻^3^, pH from 7.67 to 8.40, carbon content from 5.94% to 6.89%, and phosphorus up to 14.8 g L⁻^1^. The decreasing concentration order in soils was chromium (Cr) > nickel (Ni) > copper (Cu) > lead (Pb) > cobalt (Co) > arsenic (As) > cadmium (Cd). The soil and sediment samples showed contamination by Ni, Cu, and Cd. Analyzing the impact of solid waste dumping in the Caribbean region directly contributes to the goal of promoting more sustainable cities, ensuring the conservation of terrestrial ecosystems. Finally, this study provides key information to strengthen environmental policies and sustainable solid waste management strategies in coordination with local stakeholders and territorial authorities. The results may serve as a baseline for monitoring, prevention, and mitigation programs related to contamination by potentially toxic elements, while also contributing to the strengthening of environmental governance and the protection of ecosystems and public health.

**Supplementary Information:**

The online version contains supplementary material available at 10.1007/s11356-026-37862-1.

## Introduction

Inadequate SW management is a global problem that significantly impacts rural areas, which support the economic activities of the primary sector. This situation affects the health and quality of life of populations in these environments. This has negative impacts on developing countries, causing concern in both large cities and smaller communities (Cruvinel et al. 2019). It is estimated that the generation of municipal SW will increase from 2.1 billion tons in 2023 to 3.8 billion tons by 2050. In 2020, the global direct cost of waste management was estimated at USD 252 billion. However, when indirect costs associated with pollution, health impacts, and climate change resulting from inadequate disposal practices are considered, this figure rises to USD 361 billion. Without the implementation of urgent waste management measures, the global annual cost could nearly double by 2050, reaching approximately USD 640.3 billion (UNEP, 2024). The SW, when accumulated and exposed to direct contact with soils without any type of treatment, classification, and without a leachate evacuation system, produces harmful substances that cause the loss of minerals and increase the levels of toxicity in the soil (Choundhary et al. 2024). This leads to problems such as desertification, agricultural unproductivity, and infiltration of leachates that contaminate surface and groundwater, impairing food security (Yadav et al. 2024). This progressive deterioration without interventions makes it difficult to recover the initial conditions of the terrestrial matrix (soil).

To safeguard the environment and natural resources, as well as to fulfill the responsibility that comes with their preservation, it is imperative to address stressors in natural environments. These include population growth and population concentration in urban and rural areas. Moreover, the development of the industrial and business sectors, changes in consumption patterns, and rising standards of living, among other factors, are directly related to the increase in SW generation and the contamination of ecosystems (Sáez and Urdaneta 2014). The inadequate management of SW has significant repercussions in environmental, social, and cultural terms, which puts biodiversity at risk and therefore the quality of life of human beings. This problem is aggravated in developing countries, where less than 30% of the domestic waste produced receives adequate treatment (Cruvinel et al. 2019).

Consequently, the management of SW represents one of the main environmental challenges in Colombian cities (Vesga-Ferreira et al. 2024). During the rainy season, rain water mixes with solid waste, generating leachates that infiltrate into aquifers and soil layers. These leachates can contain a wide variety of components, such as organic content, emerging pollutants, and inorganic pollutants (Sarkar et al. 2024). In addition to the general leaching from improperly managed waste, a particularly concerning practice is OD, the illegal disposal of waste in undesignated areas such as open fields, waterways, or uncontrolled piles. The OD sites often contain a complex mixture of organic fractions and contaminants that, when mobilized through leachate and runoff, compromise soil structure and stability and may reach aquatic ecosystems and other natural resources. The constant accumulation of waste from various communities, without any environmental or sanitary control, contributes to the elevation of pollutant concentrations, including potentially toxic elements (PTEs) (Ali et al. 2019). The PTEs can be persistent in the environment and be incorporated into various matrices and the food chain (Osorio-Martínez et al. 2023; Queiros et al. 2019; Palacios-Torres et al. 2018). Their accumulation and absorption can cause toxic effects for humans and wildlife (Osorio-Martínez et al. 2023). Additionally, several PTEs are classified as endocrine disruptors, with the potential to cause alterations in endocrine functions, neurotoxicity, and DNA damage, among other effects (De la Parra-Guerra and Acevedo-Barrios, 2023; Ali et al. 2019; Rahman et al. 2019).

In South America, waste management poses an environmental challenge (Hettiarachchi et al. 2018). The waste disposal in OD lacks adequate technical and sanitary specifications, generating pollution, risks to the ecosystem, and adverse effects on biodiversity. These sites have also been linked to negative impacts on the health of surrounding populations, evidencing problems such as fatigue, allergies, psychological stress, and possible risks of congenital malformations and cancer (De Titto and Savino 2024). In 2020, Colombia generated 32,580 tons of solid waste per day, amounting to an annual total of 11,891,700 tons. Of this amount, only 1,903,269 tons (approximately 17%) were recovered (Ministry of Environment and Sustainable Development of Colombia 2022). This means that 83% of the waste is not being treated. By contrast, waste management models implemented in countries such as Switzerland, Sweden, Austria, Germany, Belgium, and the Netherlands have achieved recycling rates that significantly exceed 50%. The poor control over waste disposal in underdeveloped countries reflects the lack of attention from government entities and the academic and research community (Abubakar et al. 2022). This lack of proper waste management results in several adverse effects on soil quality and ecosystem health (Choudhary et al. 2024). In this sense, it is essential to fully understand the conditions of the soils affected to guide the formulation of environmental strategies for the effective management of SW (Liu and Hung 2023). This implies developing efficient management plans in collaboration with the affected communities, considering territorial inclusion, evaluating the impacts generated, and contributing to the priority points for decision makers and researchers (Mujtaba et al. 2024).

However, despite the recognized environmental burden of open-air dumping, evidence remains limited for TD-F landscapes. Most studies on open dumpsites have focused either on physicochemical soil alteration, metal accumulation, or ecological risk indices, whereas fewer assessments integrate chemical contamination, biological toxicity, and site-specific risk assessment in TD-F environments (Mavakala, et al. 2022). This gap is particularly relevant because tropical dry forests are seasonally water-limited ecosystems where runoff pulses, sparse vegetation cover, soil exposure, and proximity to wetlands or agricultural areas may intensify the mobilization of pollutants from unmanaged waste deposits. Therefore, open-air dumping in these landscapes should not be evaluated solely as a soil-quality problem, but as a coupled soil–sediment–biota issue requiring integrated chemical and ecotoxicological evidence.

In recent years, the use of biological models has become important for assessing the ecotoxicological risks posed by soil contamination. Among these, the nematode *Caenorhabditis elegans* has been widely adopted due to its small size, transparency, short life cycle; one of its great advantages is that its genome has between 60 and 80% similarity to humans and a well-characterized physiology (Olivero-Verbel et al. 2021; Osorio-Martínez et al. 2023). All these characteristics qualify it as an excellent biological model in the scientific field (Olivero-Verbel et al. 2021; Osorio-Martínez et al. 2023). Its sensitivity to environmental stressors makes it a valuable tool for measuring sublethal and lethal effects associated with exposure to PTEs and complex pollutant mixtures. Standardized protocols have been developed to assess endpoints such as lethality, locomotion, and growth, providing reproducible and cost-effective results in environmental monitoring and soil quality evaluations (Queirós et al. 2019; De la Parra-Guerra and Olivero-Verbel 2020).

In this context, the objective of this study was to assess soil and sediment toxicity associated with the open-air dumping of municipal SW in a dry tropical forest environment in the Colombian Caribbean. To this end, the study combined physicochemical soil characterization, quantification of potentially toxic elements via ICP-MS, multiple ecological risk indices, and toxicity bioassays using *C. elegans* as a biological model. This makes this research a novel application of a combined chemical-ecotoxicological approach applied to unmanaged waste disposal sites in dry tropical environments in Colombia. This integrated framework allows for the identification of critical contamination points, the interpretation of biological responses beyond metal concentrations alone, and the generation of evidence useful for local remediation and environmental management decisions.

## Materials and methods

### Study area

**Fig. 1:**
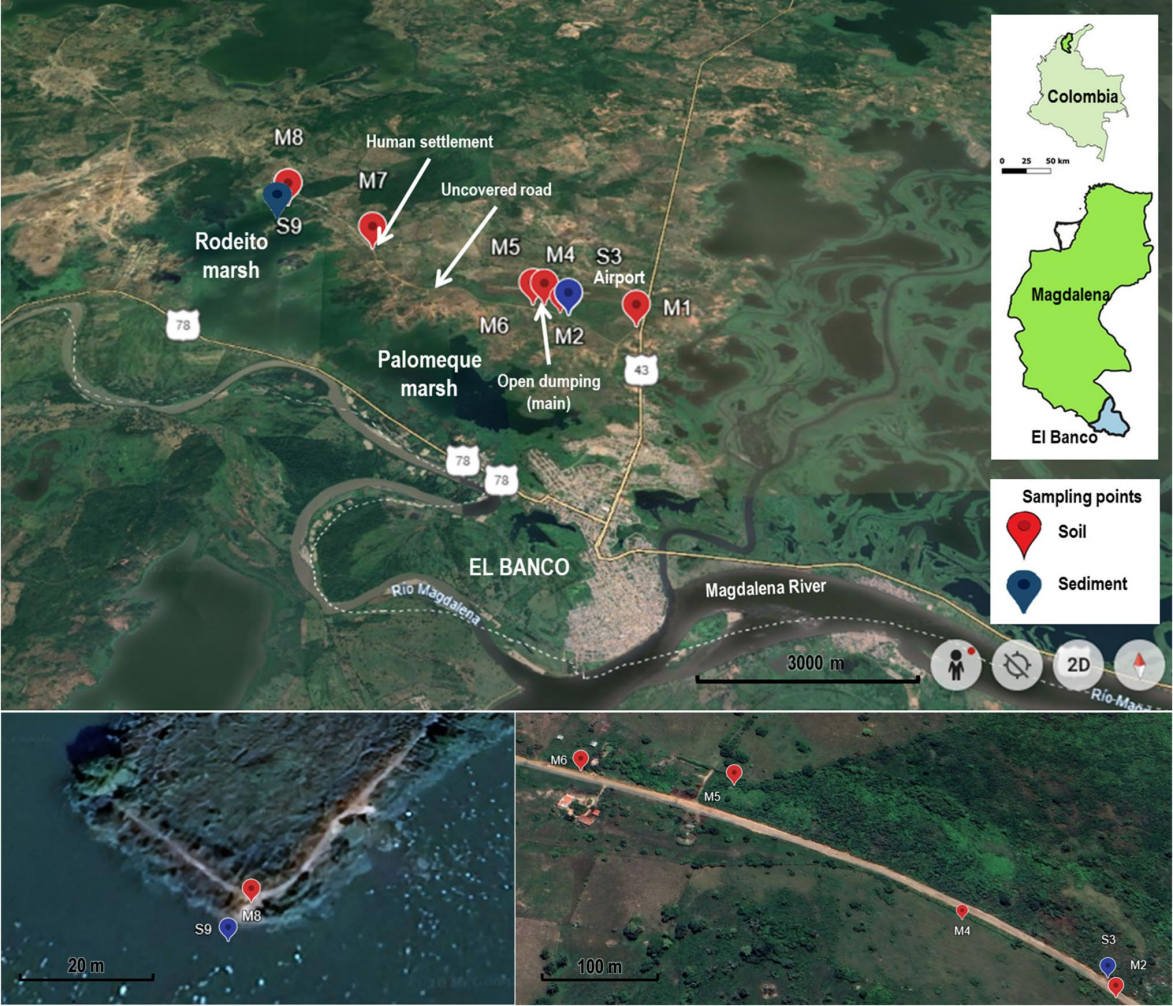
Location of the OD and sampling points. Department of Magdalena, Caribbean region of Colombia Source: Own elaboration

The municipality of El Banco covers an area of 820 km^2^ and is located in the extreme south of the Magdalena department. This region is bathed by the Magdalena and Cesar rivers, as well as by various flood zones. It has a generally flat topography marked by the Cabrito hill, with a maximum elevation exceeding 280 m above mean sea level (Fig. 1). This environment is surrounded by a complex of aquatic ecosystems that make up its geography, while the urban area exhibits a significant percentage of arboreal vegetation. The climate, influenced by the geographical position and relief, has an average temperature of approximately 35 °C. The local economy is mainly based on fishing and agriculture (Ministry of Agriculture and Rural Development of Colombia 2023).

The study was carried out in a Td-F environment in the municipality of El Banco, Magdalena department, Caribbean region of Colombia. The OD was the first of 18 landfills in this department; it is located on the “La Molina” lot, which covers an area of 4.6 hectares and is situated approximately 6 km from the urban area. At this site, located within the La Molina lot, the Public Services Company disposed of waste collected from the municipal capital. This area had previously functioned as an informal waste disposal site from 1990 to 2021. Various types of domestic waste (bags, glass bottles, cardboard, food, hygiene products), industrial waste (oils, metals, wood), and special waste (debris, soil, steel) were deposited at this site, these being the main causes of the negative environmental impacts in the area. An average of 38 tons of garbage were generated daily in this place, violating current regulations and presenting a 0% utilization rate (García, 2018).

### Soil and sediment sampling

Soil samples were collected using the OD and the nearest body of water as a reference. Sampling points were established by following the path of surface runoff toward the Rodeito Marsh. The soil was scraped superficially, removing the vegetal layer and the first two cm of the surface. A hole was made approximately 25 × 25 cm on each side and 20 cm deep (sample mass of about 1 kg) (Akanchise et al. 2020; Ahammed et al. 2024). Sediment was collected with a small shovel at points where runoff water stagnates. The samples were placed in hermetically sealed bags, labeled, and transported to the Ecotoxicology laboratory of the Corporacion Universitaria de la Costa for later analysis. All sampling points were georeferenced using a Garmin eTrex® GPS (Garmin International, Inc., USA) and are shown in Supplementary Table S1.

### Physicochemical variables

Soil and sediment samples were evaluated using the following physicochemical parameters: color using the Munsell soil table; texture according to the Bouyoucos method (Colombian Technical Standard NTC 6299); apparent density (g/cm^3^) cylinder method; true density pycnometer method; and pH potentiometric method. In addition, total organic carbon (TOC) (NTC 5403), total nitrogen (NTC 5889), and total phosphorus (NTC 6259) were determined (Trujillo 2023).

### Trace elements

Samples with a particle size of less than 100 µm were subjected to microwave-assisted wet digestion (MA-WD) following United States Environmental Protection Agency (USEPA) method 3052 (USEPA 1996). Digests were filtered and diluted to 25 mL with high-purity water. The content of trace elements and/or PTEs in the soil samples was determined using the inductively coupled plasma mass spectrometry (ICP-MS, NexION 300X model, PerkinElmer, USA), following the proposed methodology by Osorio-Martínez et al. (2021) and Santos et al. (2019). Multi-element standard solutions (10 mg L^−1^, PlasmaCal calibration solution, SCP33MS, SCP Science, Quebec, Canada) were used to prepare the calibration curve (0.1–10 μg L^−1^). Accuracy was measured using Certified Reference Materials BCR 320 and PACS-2, recovery percentages are presented in Table S2A. The calibration of the equipment was monitored by measuring the 5% HNO_3_ solution (blank) and standard solutions of known concentration every three samples. The samples were analyzed in triplicate. With the concentrations of the PTEs found, the environmental risk of the same will be established using equations and reference concentrations for soil. Limits of detection (LOD) and quantification (LOQ) are shown in Supplementary Table S2B**.**

The environmental risk associated with PTEs was assessed using multiple indices. Specifically, the contamination factor (Cf) (MacDonald et al. 2000), degree of contamination (mC_d_) (Lisiewicz et al. 2000; Wang et al. 2017), pollution load index (PLI) (Priju and Narayana 2006), ecological risk index (Eᵢʳ) (Hakanson 1980), and potential ecological risk index (RI) (Zhao et al. 2015) were used. The equations, interpretation criteria, and classification thresholds for each index are presented in Supplementary Table S3.

The reference or background concentrations for the various metals were as follows: As 4.40 μg g^−1^, Cd 0.37 μg g^−1^, Co 5.50 μg g^−1^, Cr 47.0 μg g^−1^, Cu 13.0 μg g^−1^, Ni 13.0 μgg^−1^, and Pb 22.0 μg g^−1^ (Davies 1985; Kabata-Pendias 2000). These values represent the average concentrations of each metal determined in the Earth’s crust without significant anthropogenic influence.

### Toxicity assessment

Extract preparation and nematode synchronization*.* A mixture of 10 g of soil and 20 mL of K-medium (53 mM NaCl, 32 mM potassium chloride—KCl) was prepared (Williams and Dusenbery 1990). After 24 h of mixing, the mixture was filtered and centrifuged. The extracts obtained were stored at 4 °C. Medium K was used as a control in all bioassays. Regarding the model, the wild strain N2 of *C. elegans* was used to assess the lethality, growth, and locomotion. The nematodes were preserved in K agar medium, which was prepared with KCl, sodium chloride (NaCl), agar, peptone, cholesterol, calcium chloride (CaCl_2_), and magnesium sulphate (MgSO_4_), with a bacterial lawn (*Escherichia coli*-OP50) as a food source, incubated at 20 °C (refrigerated incubator, Memmert brand). The synchronization process to have the same larval stage consisted of eliminating the adult nematodes and leaving only eggs. This was done using a bleaching solution with 0.5 mol L^−1^ sodium hydroxide (NaOH) and 0.8% hypochlorous acid (HClO), which allowed the oxidation of the adult organisms, leaving only the eggs, which have an envelope resistant to these oxidation conditions (Brenner 1974; De la Parra-Guerra and Olivero-Verbel 2020; Olivero-Verbel et al. 2021; Osorio-Martínez et al. 2023). The isolated eggs were transferred to fresh agar plates and incubated for approximately 28 h. This allowed the population to reach the fourth larval (L4) stage concurrently, ensuring synchronized individuals for the subsequent toxicological experiments**.**

#### Lethality

It was performed at the L4 larval stage, 10 ± 1 nematodes were exposed to soil and sediment extracts for 24 h at 20 °C. Death was assumed when there was no movement for a period of 30 s. This experiment was done twice with four independent replicates each (De la Parra-Guerra and Olivero-Verbel 2020; Olivero-Verbel et al. 2021; Osorio-Martínez et al. 2023)*.*

#### Locomotion

 Nematodes synchronized to the L4 larval stage, 10 ± 1 nematodes were exposed to soil and sediment extracts for 24 h, at 20 °C. For each treatment, the number of forward sinusoidal movements was recorded in 20 s intervals using a Leica dissection microscope. This experiment was performed twice with four independent repetitions each (De la Parra-Guerra and Olivero-Verbel 2020; Olivero-Verbel et al. 2021; Osorio-Martínez et al. 2023).

#### Growth

Nematodes at the L1 larval stage were used, and exposed to extracts obtained from soil and sediment for 72 h at 20 °C. For this essay, *E. coli OP50* was inoculated daily as food. The total length of the nematodes was measured using an optical microscope and with the help of ImageJ software. These measurements allowed calculating the changes in the lengths of the exposed organisms with respect to control (De la Parra-Guerra and Olivero-Verbel 2020; Olivero-Verbel et al. 2021; Osorio-Martínez et al. 2023). Approximately 30 nematodes were examined per treatment. Each treatment was evaluated three times (Tejeda-Benitez et al. 2016; Osorio-Martínez et al. 2023).

### Data analysis

Data are presented as mean ± standard error. Normal distribution and homoscedasticity were checked using the Shapiro–Wilk and Bartlett tests, respectively. Significant differences between means were determined with ANOVA/Kruskal–Wallis. Spearman correlation was performed to determine associations between variables. Statistical analyses were performed with GraphPad Prism 5 for Windows, version 5.01 (GraphPad Software, San Diego, CA) and IBM SPSS Statistics version 25 (IBM SPSS Statistics, NY, USA). The significance criterion was set at *p* < 0.05.

## Results and discussions

### Physicochemical variables of the soil

Physicochemical characteristics of the soils according to texture and relief. The soil samples collected in the OD exhibited predominantly brown colorations, with Munsell hues ranging from 5 to 10YR, commonly associated with reddish-brown to yellowish-brown soils. According to the Munsell Soil Color System, these hues generally correspond to moderate-to-dark brown tonalities with intermediate chroma and value conditions (Munsell Color Company 2009). Darker brown shades may indicate the accumulation of OM derived from the decomposition of urban solid waste, whereas reddish or yellowish tones may reflect the presence and oxidation of iron-bearing minerals promoted by leachate infiltration and fluctuating redox conditions (Brady and Weil 2016). In addition, these samples were associated with sandy-textured soils presenting relatively high bulk density values (>1.6 g cm⁻^3^), suggesting close particle packing and limited pore space. The measured particle density ranged from 2.0 to 2.44 g cm⁻^3^, values commonly associated with mineral soils containing variable proportions of quartz and silicate materials (Brady and Weil 2016**).**

The pH of soil and sediments ranged from 7.67 to 8.40. When soil pH is close to neutral or alkaline (pH ≥ 6.5), the abundance of OH- ions leads to the precipitation of insoluble compounds of Fe, Mn, Cu, and Zn. As a result, these micronutrients become unavailable for uptake by plant roots (Havlin et al. 2014; Neina 2019). In addition, the soil exchange complex becomes saturated, and the excess of Ca in the medium hinders the uptake of other elements, such as Fe, by plants (Havlin et al. 2014). This can complicate the development of agricultural activities aimed at food production.

OM contents in the samples ranged between 3 and 8%. Sample M1 had the highest OM content with 8.70%, while M6 had the lowest with 3.55%. These values suggest that the OM percentage is classified in levels ranging from moderate to very high, indicating a high OM content in the soils. However, this accumulation can generate stress situations in the vegetation. The increase in OM can clog macropores, reducing the rate of oxygen diffusion (Brady and Weil 2016). This can stress the roots, affecting their ability to absorb water due to the decrease in respiration and the energy needed for this process (Brady and Weil 2016).

The analyzed soil showed a TOC percentage ranging from 5.94% to 6.89%, values characteristic of forest soil, which usually contains broad TOC ranges depending on vegetation cover, climatic conditions, and decomposition dynamics (Pan et al. 2011; Lal 2020). Regarding total nitrogen, moderate and low levels were found. Sample M1 presented a moderate level of total nitrogen, with a value of 0.16 g mL^−1^, while M7 showed a low level of total nitrogen (about 0.04 g mL^−1^). Soils with low levels of total nitrogen are usually deficient in available nitrogen, which can affect root growth and result in stunted stem growth, leading to a low stem/root ratio (Marschner and Rengel 2012). Regarding phosphorus, most samples exhibited elevated levels. Specifically, M8 (sediment from point 1) and M2 stood out for having the highest and lowest amounts of total phosphorus, respectively, compared to the other sampling points. When the soil pH was measured, significant differences were identified. The sample with the highest phosphorus content (14.8 g L^−1^) had a pH close to neutral, while the sample with the lowest phosphorus content (2.33 g L^−1^) had an alkaline pH.

Although no significant differences were observed between the physicochemical values, correlations were identified between certain parameters. OM showed a positive correlation with nitrogen (0.725). Nitrogen, in turn, was negatively correlated with true density (−0.781). However, pH did not show statistically significant associations with the determined elements in the performed analyses.

### Potentially toxic elements in soil

The results of the descriptive statistics of the concentrations (µg g^−1^) of trace elements in soil and sediment samples are presented in Table 1.

**Table 1:** Concentration of PTEs (µg g^−1^) in soil and sediment samples from the OD in the El Banco municipality (Magdalena)

Sample type	PTEs	Minimum	Maximum	Mean	Std. deviation	Coefficient of variation (%)
**Soils**	**As**	2.16	6.40	4.02	1.61	40.10
	**Cd**	0.12	0.59	0.23	0.16	70.40
	**Co**	1.80	10.90	4.47	3.09	69.20
	**Cr**	25.00	67.00	47.60	15.40	32.40
	**Cu**	11.10	36.80	23.30	10.40	44.50
	**Ni**	28.10	43.70	31.50	5.48	17.40
	**Pb**	4.20	17.20	9.83	4.84	49.20
**Sediments**	**As**	5.80	6.61	6.22	0.55	8.87
	**Cd**	0.60	1.12	0.88	0.33	37.60
	**Co**	6.20	8.49	7.38	1.58	21.40
	**Cr**	50.80	65.80	58.30	10.60	18.20
	**Cu**	24.70	74.41	49.60	35.10	70.90
	**Ni**	37.10	44.51	40.80	5.23	12.80
	**Pb**	14.00	26.70	20.40	8.98	44.10

As shown in Table 1, in soils, the decreasing order of average concentrations is Cr > Ni > Cu > Pb > Co > As > Cd. The highest values of Cr and Ni suggest the possible presence of metallic materials or industrial residues. Ni has a low coefficient of variation (17.4%), indicating a relatively homogeneous distribution, whereas Cr shows moderate variability (32.4%), possibly associated with localized contaminant inputs. Cd and Co exhibit the highest coefficients of variation (70.4% and 69.2%), evidence of a heterogeneous distribution likely linked to dispersed or intermittent pollution sources. As (40.1%), Pb (49.2%), and Cu (44.5%) show coefficients of variation between 40 and 50%, indicating intermediate behavior in the dispersion of their concentrations. These results highlight differences in the spatial distribution of PTEs, where some metals, such as Ni, display greater uniformity, while others, such as Cd and Co, are more irregularly distributed.

The presence of PTEs in landfills is associated with industrial and vehicular waste, which serve as the main sources of contamination in the area. The high concentrations detected in landfill soils confirm the influence of these wastes. When a landfill is located near water bodies, such as streams or rivers, there is a risk of compromising environmental quality and ecological balance (Ebrahimi and Taherianfard 2011). Therefore, it is recommended that waste management practices promote awareness among residents and local authorities regarding the environmental impacts generated (Regmi et al. 2022).

For sediments, the decreasing order of concentrations is Cr > Cu > Ni > Pb > Co > As > Cd. Chromium has the highest concentration with moderate variability (18.2%), suggesting relatively uniform inputs compared to other metals. Copper, although ranking second in concentration, showed a very high coefficient of variation (70.9%), indicating marked heterogeneity in its distribution, which could be attributed to the variable input sources. Nickel has a low coefficient of variation (12.8%), reflecting a homogeneous distribution.

Lead (44.1%) and Cd (37.6%) exhibited an intermediate dispersion, while Co (21.4%) presented a more uniform distribution. Finally, As stands out for its low variability (8.87%), indicating stable concentrations in the analyzed sediments. These results show that, while some elements such as Ni and As are more uniformly distributed, others, like Cu and Pb, exhibit considerable dispersion within the study area.

The concentrations of PTEs found in this study were evaluated in relation to data reported in recent investigations conducted in various countries under comparable conditions influenced by anthropogenic activities (Table 2). In soils, the levels of Cr, Cu, and Pb were lower than those reported by Mouhoun et al. (2019) in Algeria, Mekonnen et al. (2020) in Ethiopia, and Naeem et al. (2025) in Pakistan, where considerably higher concentrations have been documented, particularly in areas with greater anthropogenic pressure. These metals were also slightly lower than those reported in Ghana (Akanchise et al. 2020), with Pb values close to those recorded in that region. These findings indicate a moderate influence of open solid waste dumping on the soils evaluated in Colombia.

**Table 2:** Comparison of PTEs in soils and sediments from different regions worldwide

Sample type	Region	As	Cd	Co	Cr	Cu	Ni	Pb	References
**Soils**	Algeria	-	1.60	-	98.90	80.10	42.10	60.40	Mouhoun-Chouaki et al. (2019)
	Ethopia	-	2.26	-	-	286.11	-	57.56	Mekonnen et al. (2020)
	Zimbabwe	-	0.40	-	41.90	130.00	-	-	Makuleke and Ngole-Jeme (2020)
	Kumasi-Ghana	4.20	8.90	-	67.00	32.00	22.00	11.00	Akanchise et al. (2020)
	Sangrampur-India	-	13.37	19.09	20.79	415.43	59.78	53.17	Dutta et al. (2022)
	Baglung-Nepal	-	10.37	-	24.23	254.54	32.91	64.23	Regmi et al. (2022)
	Bangladesh	-	4.78	4.79	6.80	46.50	10.50	422.40	Ahammed et al. (2024)
	China	101.01	2.17	-	-	116.03	-	-	Yin et al. (2024)
	Nagpur-India	-	1.00	23.90	50.35	334.10	33.05	56.70	Drall et al. (2025)
	Rawalpindi-Pakistan	-	45.12	-	436.60	447.40	248.90	909.00	Naeem et al. (2025)
	Colombia	4.02	0.23	4.47	47.60	23.30	31.50	9.83	This study (2025)
**Sediments**	Colombia	-	0.42	-	-	3.40	-	1.50	Fuentes-Gandara et al. (2021)
	Nigeria	12.00	0.71	-	-	-	18.00	11.00	Ezewudo et al. (2023)
	Bangladesh	-	-	-	64.90	1.02	-	7.90	Rahman et al. (2022)
	India	-	7.76	-	8.47	5.52	9.44	47.95	Antony et al. (2022)
	Bangladesh	2.90	4.10	-	30.20	16.90	49.00	17.60	Islam et al. (2023)
	Cuba	0.23	0.10	2.11	7.35	14.82	2.53	5.65	Bernal et al. (2024)
	Bangladesh	-	-	-	27.04	34.34	190.5	114.65	Choudhury et al. (2025)
	Mexico	4.50	-	-	7.88	6.02	-	0.91	Rendón-Von Osten et al (2025)
	Colombia	6.22	0.88	7.38	58.3	49.6.3	40.80	20.4	This study (2025)

For Cd, the values found in this study were lower than those reported in most of the compared sites, except in Zimbabwe (Makuleke and Ngole-Jeme 2020). Particularly high Cd concentrations were reported in Sangrampur-India (Dutta et al. 2022), and Baglung-Nepal (Regmi et al. 2022), exceeding the levels observed in Colombia by more than an order of magnitude. The concentrations of Co and Ni in this study were lower than those reported in other regions of the world (Dutta et al. 2022; Drall et al. 2025), although the Ni values were higher than those recorded in Ghana (Akanchise et al. 2020) and Bangladesh (Ahammed et al. 2024).

Regarding As, the value recorded in this study (4.02 µg g^−1^) was comparable to that reported by Akanchise et al. (2020) in Kumasi-Ghana (4.2 µg g^−1^), and notably lower than the value reported in China (Yin et al. 2024), which exceeded 100 µg kg^−1^. Although the concentrations reported here do not reach the extremely high levels observed in Rawalpindi, Pakistan (Naeem et al. 2025), they still indicate the influence of open dumping of solid waste, particularly due to the presence of Pb and As, which require continuous monitoring and preventive remediation strategies.

For sediments, Cr and Cu concentrations were higher than those reported in Cuba (Bernal et al. 2024), Mexico (Rendón-von Osten et al. 2025), and Bangladesh (Choudhury et al. 2025). While Pb levels exceeded those recorded in Cuba (Bernal et al. 2024) and Colombia (Fuentes-Gandara et al. 2021), they were also substantially lower than those documented in Bangladesh (Choudhury et al. 2025). Cd showed intermediate concentrations, being lower than the values reported in India (Antony et al. 2022) and Bangladesh (Islam et al. 2023), but higher than those measured in Latin American countries (Fuentes-Gandara et al. 2021; Bernal et al. 2024). For Co, in this study, the levels were 3.5 times higher compared to those reported for Cuba (Bernal et al. 2024). Regarding Ni, the concentrations were higher than those observed in Nigeria (Ezewudo et al. 2023), India (Antony et al. 2022), and Cuba (Bernal et al. 2024); yet they remained below the levels reported for Bangladesh (Islam et al. 2023; Choudhury et al. 2025).

Overall, the results show that the soils and sediments from the study area present intermediate PTE concentrations compared to international data. However, Cr, Cu, and Pb stand out for exhibiting relatively high levels when contrasted with several reference regions, highlighting the influence of anthropogenic inputs and the need for ongoing environmental monitoring.

### Environmental risk of PTEs in soil and sediments

The results of the indices used in the potential ecological risk assessment, such as Cf, mCd, Eᵢʳ, PLI, and RI, are presented in Table 3. According to the Cf, Ni showed values above 2 at all sampling points, indicating moderate contamination, and in M4 and S9, even strong contamination (Cf ≥ 3). This trend may be associated with the fact that both sites are exposed to sources of solid waste: M4 is located at a dumpsite along an unpaved road, while S9 is situated on the shore of the Rodeito marsh, where contaminants can accumulate through runoff and water transport. Cu exhibited a similar pattern, with moderate contamination at most stations and a particularly high value at M6 (5.72), a critical point on the periphery of the main open dump, suggesting the mobilization of metals by runoff. Arsenic recorded moderate contamination at M4, M5, M6, M7, and S9, while remaining at low levels at the other points. Cadmium showed moderate contamination at M8 and S9, with M6 being the most critical site, reaching a value of 3.02, consistent with its location near the main OD, being susceptible to the transport of pollutants. Co reflected moderate contamination at M6, M7, and S9, with low values at the remaining sites. Cr presented values close to 1, indicating moderate contamination at most stations, except for M1 and M2, whose locations are far from direct waste sources, which may explain their lower levels. Lead did not show concerning concentrations, except at M6, where moderate contamination was recorded. Several studies have reported that the moderate Cf in soil and sediments for Cr, Ni, As, Cd, and Pb is likely associated with sources such as oil leaks, vehicle component wear, the use of metal alloys, foundry activities, electronic component manufacturing, and chemical industry processes (Höss et al. 2009; Kormoker et al. 2019; Islam et al. 2023).

**Table 3:**
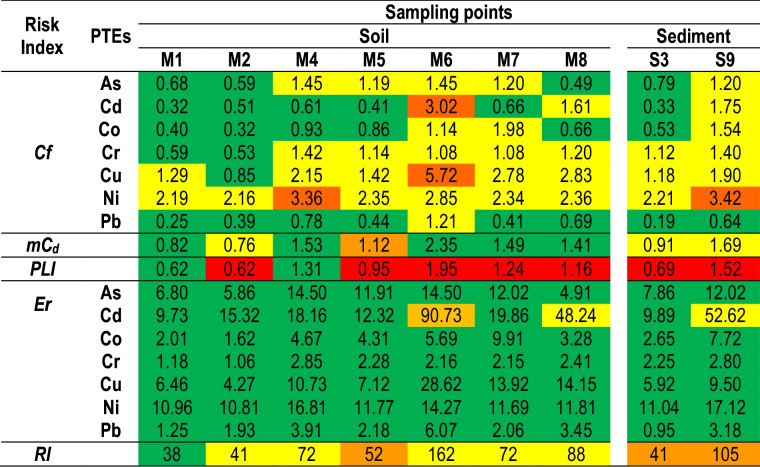
Calculations of pollution indices and ecological risk by PTEs in soil and sediment of the OD in the El Banco municipality (Magdalena). “M” corresponds to soil sampling points, whereas “S” represents sediment sampling points

*Classification criteria: Not contaminated (green); Moderate (yellow); Strong (orange); Very strong; Extreme (red) (SQGs)

The accumulation of toxic elements in sediments is an important factor in the self-purification of aquatic ecosystems. However, when this process is reversible, it can constitute a significant and constant source of secondary pollution, particularly in periods where water availability is scarce and the water column is reduced (Linnik and Zubenko 2002). Waste from industrial sources can endanger human and environmental health. Mining can cause alterations in the soil and subsoil, such as infertility, and superficial removal of soil, allowing the passage of contaminants through water or by direct incorporation into organic levels (Dehkordi et al. 2024). In some cases, contamination is the result of improper application, handling, or disposal of toxic substances. Spills of chemical substances such as motor oil, cooking oil, pesticides, petroleum, and solvents, among others, cause contamination in the soil and groundwater (Elijah 2022).

The mC_d_ indicated that most sampling points were not contaminated (mC_d_ < 1.5). However, M4 and S9 presented slight contamination (1.5 < mC_d_ < 2.0), which is related to their exposure to solid waste and water bodies. At M6, the value reached 2.35, classifying it as moderately contaminated (2 < mC_d_ < 4). These results suggest that sites linked to water bodies and waste disposal areas present greater accumulation of trace elements, likely due to external inputs and contaminant transport processes.

The PLI showed that M4, M6, M7, M8, and S9 recorded values above 1, reflecting contaminated conditions. The highest impact was observed at M6 (1.95), a site where landfill leachate and runoff converge. Under tropical environmental conditions, elevated temperatures and seasonal humidity may accelerate the decomposition of organic residues and enhance leachate generation, promoting the release and redistribution of PTEs within the soil matrix. In addition, the elevated OM content at convergence zones such as M6 may influence metal behavior through adsorption, complexation, and temporary sequestration processes, while the decomposition of OM may also facilitate metal mobilization through soluble organic-metal complexes. These combined processes may contribute to the accumulation and persistence of contaminants at sites directly influenced by runoff and waste disposal activities. In contrast, M1, M2, M5, and S3 presented values below 1, suggesting the absence of significant contamination and less critical conditions, consistent with their lower exposure to direct waste sources.

Regarding the individual Eᵢʳ, Cd was the metal with the highest contribution to risk, reaching considerable values (80 ≤ Eᵢʳ < 160) at M6 (90.73) and moderate values (40 ≤ Eᵢʳ < 80) at S9 (52.62) and M8 (48.24). These results are explained by the proximity of M6 and M8 to active waste sources, as well as the location of S9 on the lagoon shore, where contaminants tend to accumulate. Islam et al. (2023) and Bernal et al. (2024) also reported that Cd had the greatest contribution to biological hazards in sediments collected from rivers. It is well known that phosphate fertilizer and pesticide manufacturing companies are major producers of various toxic substances, including Cd (Su et al. 2013). Several studies have indicated that the increase in Cd concentrations is mainly driven by human activities (Aminiyan et al. 2018; Kamani et al. 2017). Cu also contributed significantly to the risk at M6 (28.62), while Ni exhibited low risk levels at all stations. The remaining metals (As, Co, Cr, and Pb) generally posed low ecological risks. It is worth noting that the Eᵢʳ for the soil samples in this study was low, except for Cd at M8, which showed a moderate risk. Zhao et al. (2015) conducted an ecological risk assessment on soils from an e-waste recycling site in Southeast China and reported moderate contamination by elements such as Ni, Co, and Cd, which were identified as the main contributors to ecological risk. Finally, the potential ecological risk index (RI) revealed that M6 and S9 are at moderate risk (162 and 105, respectively), while the remaining evaluated sites showed low ecological risk. These findings are consistent with those reported by Bernal et al. (2024), who reported RI values below 150 for sediments collected in Cuba.

### Toxicity assessment

**Fig. 2:**
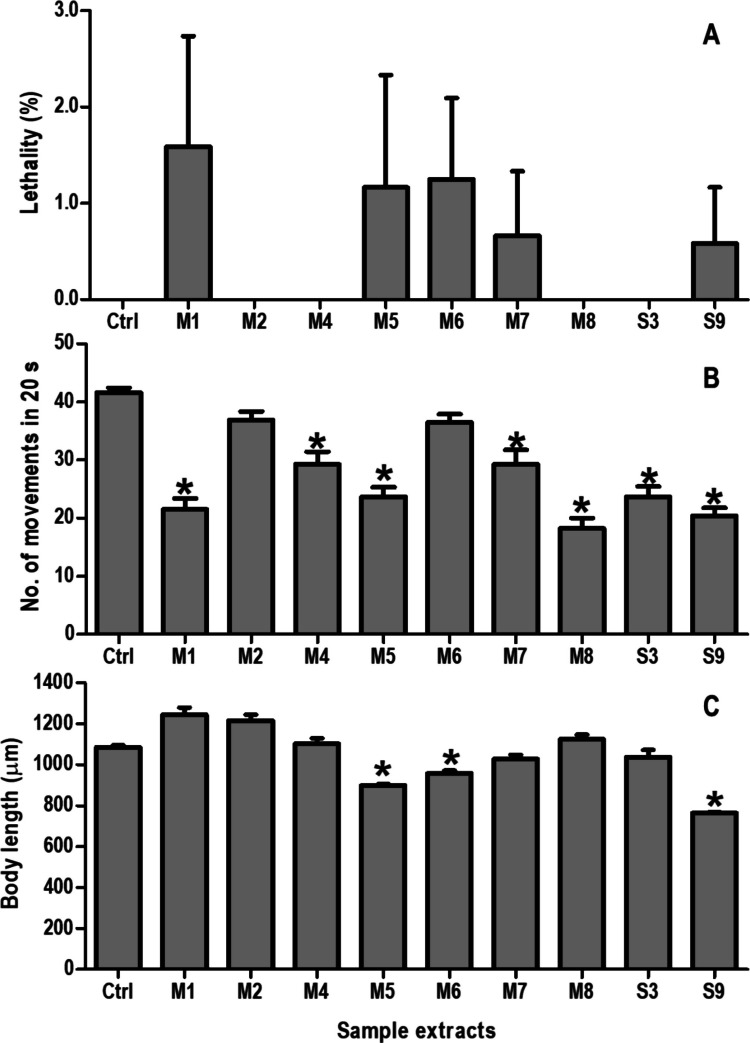
Toxicity assessment of soil and sediment extracts on *C. elegans*. **A** Lethality expressed as survival percentage, **B** locomotion expressed as body bends per 20 s, and **C** growth expressed as body length (µm). Values are presented as mean ± standard deviation

The results of the toxic effects of aqueous extracts from soil and sediment samples on *C. elegans* are presented in Fig. 2. The lethality test showed that exposure of nematodes to the different extracts did not reveal significant differences compared to the control group. The worms showed 0% lethality, which is read as 100% survival, for four of the sampling points and the control. Minimum lethality percentages of 1 and 2% were recorded in the extracts from sampling points M1, M5, M6, M7, and S9. These results indicate that the behavior of the data does not cause concern, since there is a high probability that the organism's populations are not affected by the extracts to which they were exposed (Fig. 2A). The results of the bioassays for lethality, locomotion, and growth showed no significant associations with PTEs (Table S4). Previous studies have reported that acute exposure to PTEs in dust, soil, and sediment extracts causes lethality in *C. elegans* (Valdelamar-Villegas et al. 2021; Osorio-Martinez et al. 2023). It has also been reported that both Ni and Cr reduce the survival of *C. elegans* (Rudel et al. 2013), which are the main elements in the soil and sediment samples in this study. This suggests that the concentration of the elements has not reached levels that pose a high risk to the survival of the biological model.

The assessment of toxicity on locomotion showed that exposure of nematodes to different soil and sediment extracts reduced body movements in a moderately concentration-dependent manner, showing significant differences compared to the control group. The greatest inhibition of movement was observed in M1, M5, M8, S3, and S9, reaching almost half (50%) of normal activity (Fig. 2B). In this context, the main sources of water could be identified, such as the Palomeque marsh and crops from nearby farms. PTEs such as Cd, Cr, Cu, Co, and Ni are inversely correlated with this parameter in *C. elegans* (Tejeda-Benítez et al. 2016; Osorio-Martínez, et al., 2023). In addition, binary and tertiary mixtures of trace elements can cause a large decrease in locomotion in the biological model (Moyson et al. 2018). These factors could affect both the neurological and biological aspects of nematodes.

The effect of the extracts on the growth of *C. elegans* was determined by body length, which showed that the exposed nematodes presented significant differences compared to the control group. Growth inhibition was observed in M5, M6, and S9 compared to the control, with a growth percentage of 0.7% to 0.9%, indicating toxicity assessed by the inhibition of nematode growth. In addition, a significant reduction in the body length of the nematodes was recorded in the range of 34 to 38%, associated with the contributions of agricultural activities on farms. On the other hand, a less well-defined concentration–response curve could be seen (Fig. 2C**).** The growth of *C. elegans* has been reported to be inhibited by different PTEs present in soil and river sediments (Höss et al. 2009; Tejeda-Benitez et al. 2016). Elements as Cu and Pb have presented a negative correlation with the growth of nematodes (Höss et al. 2009).

## Conclusions

The results obtained in this study showed a moderate influence of open-air solid waste disposal on the physical–chemical characteristics of the soil and the accumulation of potentially toxic elements. Significant levels of OM, total organic carbon, and phosphorus were identified, as well as an alkaline pH, conditions that can hinder the absorption of certain essential nutrients by plants. Regarding potentially toxic elements, Cd and Cu were found in significant concentrations at some locations, as reflected in the ecological risk and pollution load indices, which classify several sites as moderately or heavily contaminated. The most critical points (M6 and S9) were located near the main dump and in the Rodeito Marsh, suggesting a dispersion of contaminants from the disposal site into the surrounding environment.

On the other hand, toxicological tests performed with *C. elegans* showed relevant sublethal effects. Although mortality was practically zero, alterations in the growth and locomotion of the nematodes were observed, indicating a physiological response to chemical stress induced by soil extracts. Growth inhibition at some points can be associated with PTEs such as Cr, Cu, Co, and Ni, while the reduction in mobility may reflect possible neurotoxic effects related to the presence of metals and other potential contaminants. These findings highlight the need to implement continuous monitoring measures and remediation strategies, especially at the most affected sites, to prevent long-term risks to biota and ecosystem health.

From a management perspective, these findings provide evidence to prioritize local remediation actions at the most affected sites, particularly those located near the main dumping area and the Rodeito Marsh. Local authorities should consider immediate measures such as restriction of new waste inputs, control of runoff and leachate dispersion, physical stabilization of exposed soils, removal or isolation of highly contaminated materials, and periodic monitoring of PTEs in soils and sediments. The integration of ecological risk indices with *C. elegans* bioassays is especially valuable because it links chemical contamination with biological responses, allowing risk interpretation beyond concentration thresholds alone. Therefore, this approach can support municipal solid waste management plans, guide restoration priorities in El Banco, and serve as a transferable assessment framework for other Td-F regions facing similar open-air dumping pressures.

## Supplementary Information

Below is the link to the electronic supplementary material.

ESM 1 (DOCX 52.6 KB)

## Data Availability

The datasets generated during and/or analysed during the current study are not publicly available but are available from the corresponding author on request.
