# Supplementary material for: Impact of open-air dumping of urban solid waste on soil toxicity and properties in a tropical environment
Source: Environ Sci Pollut Res Int. 2026 Jul 3;33(21):10592–606. doi: 10.1007/s11356-026-37862-1 (PMC13369223; doi:10.1007/s11356-026-37862-1)
Supplement: Supplementary file 1 — (DOCX 52.6 KB) [file 11356_2026_37862_MOESM1_ESM.docx]

**Supplemental material**

**Impact of open-air dumping of urban solid waste on soil toxicity and properties in a tropical environment**

Ana De la Parra-Guerra; Kelly Rodelo-Soto; Shania Polo-Camargo; Mildreth Pallares-Arévalo; Érico [Flores](https://protect.checkpoint.com/v2/r01/___https://acsess.onlinelibrary.wiley.com/action/doSearch?ContribAuthorRaw=Flores%2C+Erico+M+M___.YzJ1OmN1YzpjOm86ZjBhOTQ4NGU2NWZiZDBjOGJkOTRlNGE3ZTUyYTBjMGE6Nzo2NWFjOjlmNTM5ZmRiMzBkNThlYmViYzI5Y2RhODdiNmE5NmZiNGYyYzhmZmM0ZjEwMWVjMTRjZjk5ODA4MDZjOTI5OWE6cDpUOkY); Rochele Picoloto, Cristian Andriolli, Jorge Osorio-Martínez; Katy Rematoza-Chamorro, Fabio Fuentes-Gandara

**Table S1.** Geographic coordinates of sampling points in El Banco (Magdalena).

| **Sampling points** | | **Coordinates** | | **Description** |
| --- | --- | --- | --- | --- |
|  |  | **X** | **Y** |  |
| **Soil** | **M1** | 9.03698800 | -73.96433600 | Unpaved access road approximately 50 m from the road leading to the airport. |
|  | **M2** | 9.03955500 | -73.97265800 | Open dumping on the side of an unpaved tertiary road. |
|  | **M4** | 9.04030200 | -73.97374400 | Open dumping on the side of an unpaved tertiary road. |
|  | **M5** | 9.04166100 | -73.97528200 | Inside the main open dumping. Approximately 1300 m from the Palomeque Marsh. |
|  | **M6** | 9.04182000 | -73.97702300 | Outside the main open dumping. On the unpaved tertiary road. |
|  | **M7** | 9.05508200 | -73.99719800 | Human settlement. Approximately 3100 m from the main open dumping (M5). |
|  | **M8** | 9.06580500 | -74.00931600 | Bank of the Rodeito Marsh. Approximately 2200 m from M7. |
| **Sediment** | **S3** | 9.03966300 | -73.97275400 | Open dumping on the side of an unpaved tertiary road. Seasonal stream on the side of the road. |
|  | **S9** | 9.06580500 | -74.00931600 | Sediment taken 1 m from the banks of the Rodeito Marsh. |

**Table S2A.** Quality control of analytical methods used for determination of trace elements in urban dusts. Accuracy was measured using Certified Reference Materials BCR 320 and PACS-2.

| **Element** | **BCR 320 (Channel Sediment)** | | | **PACS-2 (Marine Sediment )** | | |
| --- | --- | --- | --- | --- | --- | --- |
|  | **Certified value (µg g^-1^)*** | **Found value**  **(µg g^-1^)** | **Recovery (%)** | **Certified value**  **(µg g^-1^)** | **Found value**  **(µg g^-1^)** | **Recovery (%)** |
| V | 105 | 101 | 96.2 | 133 | 129 | 97.0 |
| Cr | 138 | 135 | 97.8 | 90.7 | 88.1 | 97.1 |
| Co | 19 | 18.1 | 95.3 | 11.5 | 10.8 | 93.9 |
| Ni | 75.2 | 74.1 | 98.5 | 39.5 | 38.9 | 98.5 |
| Cu | 44.1 | 43.2 | 97.9 | 310 | 302 | 97.4 |
| As | 76.7 | 73.4 | 95.7 | 26.2 | 25.0 | 95.4 |
| Cd | 0.533 | 0.510 | 95.7 | 2.11 | 2.01 | 95.3 |
| Pb | 42.3 | 41.1 | 97.2 | 183 | 178 | 97.3 |

*. Dry weight. The analytes (Ag. Bi. Hg. Mo. Sn. Tl. and Zn) presented values below the LOD.

**Table S2B.** Limits of Detection (LODs) and limits of quantification (LOQs) for trace element analysis in urban soil and sediment (µg g^-1^. dry weight).

| **Element** | **LOD** | **LOQ** |
| --- | --- | --- |
| Ag | 0.27 | 0.90 |
| As | 0.05 | 0.18 |
| Bi | 0.01 | 0.03 |
| Cd | 0.09 | 0.30 |
| Co | 0.08 | 0.27 |
| Cr | 0.02 | 0.07 |
| Cu | 2.03 | 6.70 |
| Hg | 0.01 | 0.04 |
| Mo | 0.10 | 0.33 |
| Ni | 0.04 | 0.13 |
| Pb | 0.04 | 0.13 |
| Sn | 0.05 | 0.17 |
| Tl | 0.06 | 0.20 |
| V | 0.04 | 0.14 |
| Zn | 0.11 | 0.36 |

**Table S3.** Indices for calculating environmental risks associated with PTEs.

| Index equation | Description | Categories | References |
| --- | --- | --- | --- |
| *Contamination factor* 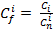 | *C_i_:* concentration of the elements in the sample.  : reported background value for soil. 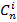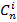 | Uncontaminated. C*_f_* < 1;  moderate contamination. 1 ≤ C*_f_* < 3;  very strong contaminated. 3 ≤ C*_f_* < 6; and  extremely contaminated when C*_f_* ≥ 6. | Hakanson, 1980; MacDonald et al., 2000; Palacios-Torres et al., 2018. |
| *Degree of soil contamination* 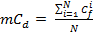 | *N:* number of elements analyzed.  *C_f :_* pollution factor. | Uncontaminated. *mC_d_* < 1.5;  slightly contaminated. 1.5 ≤ *mC_d_* < 2; moderately contaminated. 2 ≤ *mC_d_* < 4; considerably contaminated. 4 ≤ *mC_d_* < 8; highly contaminated. 8 ≤ *mC_d_* < 16;  heavily contaminated. 16 ≤ *mC_d_* < 32; and extremely contaminated. *mC_d_* > 32 | Lisiewicz et al., 2000; Wang et al., 2017. |
| *Pollution Load Index* 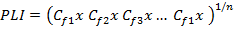 | *C_f:_* pollution factor. | Uncontaminated when *PLI* <1. and contaminated when *PLI* ≥ 1 | Priju and Narayana, 2014. |
| *Ecological risk index* 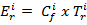 | *T_r_:* toxicity coefficient of element *i*.  The toxicity coefficients for Cr. Co. Ni. Cu. Zn. As. Cd. Hg. and Pb are 2. 5. 5. 5. 1. 10. 30. 40. and 5. respectively | Low risk. *E_r_* < 40;  moderate risk. 40 ≤ *E_r_* < 80;  considerable risk. 80 ≤ *E_r_* < 160;  high risk. 160 ≤ *E_r_* < 320; and  very high risk. *E_r_* > 320. | Hakanson, 1980. |
| *Potential ecological risk index* 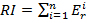 | *E^i^_r_*: Ecological risk index | Low risk. *RI* < 105;  moderate risk. 105 ≤ *RI* < 210;  considerable risk. 210 ≤ *RI* < 420; and  high risk. *RI* ≥ 420 | Zhao et al., 2015. |

**Table S4.** Spearman's correlation analysis to determine the association between the variables under study.

|  | As | Cd | Co | Cr | Cu | Ni | Pb | MO | N | C | P | DR | Lethality | Locomotion | Growth |
| --- | --- | --- | --- | --- | --- | --- | --- | --- | --- | --- | --- | --- | --- | --- | --- |
| Cd | 0.450 |  |  |  |  |  |  |  |  |  |  |  |  |  |  |
|  | 0.230 |  |  |  |  |  |  |  |  |  |  |  |  |  |  |
| Co | **0.783** | **0.683** |  |  |  |  |  |  |  |  |  |  |  |  |  |
|  | **0.017** | **0.042** |  |  |  |  |  |  |  |  |  |  |  |  |  |
| Cr | 0.500 | 0.350 | 0.433 |  |  |  |  |  |  |  |  |  |  |  |  |
|  | 0.178 | 0.356 | 0.244 |  |  |  |  |  |  |  |  |  |  |  |  |
| Cu | 0.333 | **0.783** | **0.700** | 0.383 |  |  |  |  |  |  |  |  |  |  |  |
|  | 0.385 | **0.013** | **0.036** | 0.308 |  |  |  |  |  |  |  |  |  |  |  |
| Ni | **0.733** | **0.717** | **0.717** | **0.850** | 0.667 |  |  |  |  |  |  |  |  |  |  |
|  | **0.025** | **0.030** | **0.030** | **0.004** | 0.050 |  |  |  |  |  |  |  |  |  |  |
| Pb | 0.467 | **0.800** | 0.550 | 0.567 | **0.817** | **0.817** |  |  |  |  |  |  |  |  |  |
|  | 0.205 | **0.010** | 0.125 | 0.112 | **0.007** | **0.007** |  |  |  |  |  |  |  |  |  |
| MO | **0.983** | 0.400 | **0.750** | 0.517 | 0.350 | **0.717** | 0.500 |  |  |  |  |  |  |  |  |
|  | **<0.001** | 0.286 | **0.020** | 0.154 | 0.356 | **0.030** | 0.170 |  |  |  |  |  |  |  |  |
| N | 0.536 | 0.075 | 0.603 | 0.494 | 0.377 | 0.410 | 0.293 | 0.628 |  |  |  |  |  |  |  |
|  | 0.137 | 0.847 | 0.086 | 0.177 | 0.318 | 0.273 | 0.444 | 0.070 |  |  |  |  |  |  |  |
| C | 0.350 | 0.550 | 0.650 | 0.083 | **0.717** | 0.350 | 0.650 | 0.417 | 0.569 |  |  |  |  |  |  |
|  | 0.356 | 0.125 | 0.058 | 0.831 | **0.030** | 0.356 | 0.058 | 0.265 | 0.110 |  |  |  |  |  |  |
| P | 0.150 | 0.567 | 0.433 | -0.067 | **0.733** | 0.233 | 0.667 | 0.233 | 0.335 | **0.917** |  |  |  |  |  |
|  | 0.700 | 0.112 | 0.244 | 0.865 | **0.025** | 0.546 | 0.050 | 0.546 | 0.379 | **0.001** |  |  |  |  |  |
| DR | -0.283 | 0.300 | -0.133 | -0.183 | -0.217 | -0.150 | -0.200 | -0.400 | -0.561 | -0.300 | -0.250 |  |  |  |  |
|  | 0.460 | 0.433 | 0.732 | 0.637 | 0.576 | 0.700 | 0.606 | 0.286 | 0.116 | 0.433 | 0.516 |  |  |  |  |
| Lethality | -0.502 | -0.192 | -0.548 | -0.256 | -0.475 | -0.329 | -0.237 | -0.575 | **-0.770** | -0.411 | -0.365 | 0.475 |  |  |  |
|  | 0.168 | 0.621 | 0.127 | 0.507 | 0.197 | 0.388 | 0.539 | 0.105 | **0.015** | 0.272 | 0.334 | 0.197 |  |  |  |
| Locomotion | 0.209 | -0.126 | 0.226 | 0.142 | 0.301 | 0.151 | 0.351 | 0.326 | 0.538 | 0.611 | 0.510 | **-0.828** | -0.202 |  |  |
|  | 0.589 | 0.748 | 0.559 | 0.715 | 0.431 | 0.699 | 0.354 | 0.391 | 0.135 | 0.081 | 0.160 | **0.006** | 0.603 |  |  |
| Growth | -0.068 | -0.346 | -0.270 | -0.354 | -0.338 | -0.447 | -0.245 | 0.059 | 0.237 | 0.160 | 0.262 | -0.177 | -0.282 | 0.195 |  |
|  | 0.863 | 0.362 | 0.482 | 0.349 | 0.374 | 0.227 | 0.526 | 0.880 | 0.539 | 0.680 | 0.496 | 0.648 | 0.462 | 0.615 |  |
| pH | 0.424 | 0.373 | 0.254 | -0.034 | -0.102 | 0.203 | 0.017 | 0.305 | -0.162 | -0.153 | -0.237 | 0.509 | -0.028 | -0.630 | -0.215 |
|  | 0.256 | 0.323 | 0.509 | 0.931 | 0.795 | 0.600 | 0.965 | 0.425 | 0.678 | 0.695 | 0.539 | 0.162 | 0.943 | 0.069 | 0.579 |

**References**

Hakanson L (1980) An ecological risk index for aquatic pollution control. A sedimentological approach. Water Res 14(8): 975-1001. https:// doi.org/10.1016/0043-1354(80)90143-8

MacDonald DD, Ingersoll CG, Berger TA (2000) Development and evaluation of consensus-based sediment quality guidelines for freshwater ecosystems. Arch Environ Contam Toxicol 39(1): 20-31. <https://doi.org/10.1007/s002440010075>

Palacios-Torres Y, Caballero-Gallardo K, Olivero-Verbel J (2018) Mercury pollution by gold mining in a global biodiversity hotspot, the Choco biogeographic region, Colombia. Chemosphere 193: 421-430. <https://doi.org/10.1016/j.chemosphere.2017.10.160>

Lisiewicz M, Heimburger R, Golimowski J (2000) Granulometry and the content of toxic and potentially toxic elements in vacuum-cleaner collected, indoor dusts of the city of Warsaw. Sci Total Environ 263(1-3): 69-78. <https://doi.org/10.1016/S0048-9697(00)00667-7>

Priju CP, Narayana AC (2006) Spatial and temporal variability of trace element concentrations in a tropical lagoon, Southwest Coast of India: Environmental Implications. J Coastal Res 39:1053-1057.

Wang Y, Ling M, Liu RH, Yu P, Tang AK, Luo XX, Ma Q (2017) Distribution and source identification of trace metals in the sediment of Yellow River Estuary and the adjacent Laizhou Bay. Phys Chem Earth Parts A/B/C 97: 62-70. <https://doi.org/10.1016/j.pce.2017.02.002>

Zhao W, Ding L, Gu X, Luo J, Liu Y, Guo L, Shi Y, Huang T, Cheng S (2015) Levels and ecological risk assessment of metals in soils from a typical e-waste recycling region in southeast China. Ecotoxicology 24(9): 1947-1960. <https://doi.org/10.1007/s10646-015-1532-7>
